# Cocaine induces cell death and activates the transcription nuclear factor kappa-b in pc12 cells

**DOI:** 10.1186/1756-6606-2-3

**Published:** 2009-02-01

**Authors:** Lucilia B Lepsch, Carolina D Munhoz, Elisa M Kawamoto, Lidia M Yshii, Larissa S Lima, Maria F Curi-Boaventura, Thais ML Salgado, Rui Curi, Cleopatra S Planeta, Cristoforo Scavone

**Affiliations:** 1 Department of Pharmacology Institute of Biomedical Sciences, University of São Paulo, Avenida Professor Lineu Prestes, 1524. 05508-900 – São Paulo, Brazil; 2 Department of Physiology and Biophysics, Institute of Biomedical Sciences, University of São Paulo-Brazil; 3 Laboratory of Neuropsychopharmacology School of Pharmaceutical Sciences UNESP – São Paulo State University-Brazil

## Abstract

Cocaine is a worldwide used drug and its abuse is associated with physical, psychiatric and social problems. The mechanism by which cocaine causes neurological damage is very complex and involves several neurotransmitter systems. For example, cocaine increases extracellular levels of dopamine and free radicals, and modulates several transcription factors. NF-κB is a transcription factor that regulates gene expression involved in cellular death. Our aim was to investigate the toxicity and modulation of NF-κB activity by cocaine in PC 12 cells. Treatment with cocaine (1 mM) for 24 hours induced DNA fragmentation, cellular membrane rupture and reduction of mitochondrial activity. A decrease in Bcl-2 protein and mRNA levels, and an increase in caspase 3 activity and cleavage were also observed. In addition, cocaine (after 6 hours treatment) activated the p50/p65 subunit of NF-κB complex and the pretreatment of the cells with SCH 23390, a D1 receptor antagonist, attenuated the NF-κB activation. Inhibition of NF-κB activity by using PDTC and Sodium Salicilate increased cell death caused by cocaine. These results suggest that cocaine induces cell death (apoptosis and necrosis) and activates NF-κB in PC12 cells. This activation occurs, at least partially, due to activation of D1 receptors and seems to have an anti-apoptotic effect on these cells.

## Introduction

Cocaine is a drug of abuse with a prevalence of 2.8% in USA and 0.3% worldwide. Cocaine is the second highest drug of abuse in USA, according to the United Nations Office on Drug and Crime (UNODC) (source: World Drug Report, 2007; ).

Cocaine inhibits dopamine transporter (DAT) in plasma membrane causing an increase in extracellular dopamine levels. This results in the stimulation of the brain reward pathway that can lead to the development of addiction [1,2]. Addiction is a chronic relapsing disease [3] and its treatment is the most expensive of the neuropsychiatric disorders [4] mainly due to the costs of healthcare, productivity loss and crime (Office of National Drug Control Policy, 2004; United Nations Office on Drugs and Crime, 2007).

Along with addiction, cocaine can also induce neurological impairment (deficits in cognition, motivation, insight and attention), behavioral disinhibition, emotional instability, impulsiveness, and movement disorders [5,6]. Clinical and pre-clinical studies have demonstrated the occurence of learning and memory impairment and movement disorders in cocaine abusers, even after a long period of drug withdrawal [7,8]. Although the cellular mechanisms underlying this deficit have not been identified yet, several lines of investigation suggest that either necrotic or apoptotic neuronal death may account for drug-of-abuse-induced neurological impairment [9].

Necrotic cell death involves loss of membrane integrity and selective permeability, whereas apoptotic cell death is characterized by membrane blebbing, cell shrinkage and chromatin condensation and fragmentation. The apoptotic changes are often accompanied by caspase activation and cytochrome c release into cytosol [10]. Members of the Bcl-2 family of proteins (Bax, Bak, Bcl-XL, Bcl-2, and others) regulate mitochondrial integrity and cytochrome c release [11,12] and so are important determinants of cell death or survival [13,14].

Cocaine neurotoxicity has been associated with induction of apoptosis such as activation of caspase [15-19], loss of mitochondrial potential, and cytochrome c release into cytosol [16,18], and alteration of Bax/Bcl-2 ratio [19]. Cocaine-induced expression of immediate early genes (c-fos, c-jun) and transcription factors (CREB) has been reported by several authors [20-22] and it is suggested that changes in gene transcription may contribute to the development of adaptative responses induced by the use of cocaine [23].

Nuclear factor-κB (NF-κB) is a transcription factor found in a variety of cell types including neurons and microglia [24]. NF-κB can be activated by pro-inflammatory stimuli, such as pathogen-derived lipopolysaccharide (LPS), cytokines (TNFα, IL-1β) and reactive oxygen species [25-27]. NF-κB, comprised members of Rel/NF-κB family of proteins, forming homo-and heterodimers through combination of the p65 (or RelA), p50, p52, c-Rel or RelB subunits. It is constitutively expressed in the cytoplasm where it is bound to IκB, a protein that masks the nuclear localization signal of NF-κB thereby retaining it in the cytoplasm [28]. Inducers of NF-κB act through intracellular signaling cascades that activate the IκB kinases (IKKs), which phosphorylate two specific N-terminal serines of IκBα resulting in IκBα polyubiquitination and degradation in the 26S proteasome [29]. When IκBα is degraded, NF-κB migrates to the nucleus and modulates transcription of target genes involved in cell death. To further understand cocaine-induced neurotoxicity we tested whether cocaine induces cell death (apoptosis and necrosis) and activates NF-κB in PC12 cells.

## Methods

### Reagents

Dulbecco's modified Eagle's medium (DMEM), bovine serum, horse serum, trypsin, penicillin and streptomycin were provided by Cultilab (Campinas, Brazil). Reagents for SDS-PAGE and immunoblotting were purchased from Bio-Rad Laboratories (Richmond, CA, USA). SCH23390 was from ToCris, Missouri, USA; PDTC and Sodium salicilate were obtained from Sigma-Aldrich, St Loui, MO, USA. γ-^32^P-ATP and poly dI-dC from Amersham Biosciences (Uppsala, Sweden), the gel shift assay system kit for NF-κB from Promega (Madison, WI, USA), and the BioRad protein assay kit from BioRad (Hercules, CA, USA). Routine reagents were from Sigma-Aldrich (St Louis, MO, USA).

### Preparation of PC12 cells

PC12 cells, a dopaminergic neuronal model, were maintained in DMEM supplemented with 5% heat inactivated bovine serum, 10% horse serum, penicillin (100 units/mL), streptomycin (25 units/mL). Confluent cultures were washed with phosphate-buffered saline (PBS), detached with 2.0 mM EDTA, centrifuged and subcultured (7.10^4 ^cell/well) to poly L-lysine coated 6-well plates. After 48 hours, the medium was replaced by a DMEM without serum and cultured under different combinations of time exposure periods and drug treatments.

### Treatment of cells with cocaine and inhibitors

Cells were treated for the indicated incubation periods with various concentrations of cocaine. Cells treated with PBS was used as controls. The inhibitory agents were dissolved directly into the culture medium at the indicated concentration 20 minutes before cocaine treatment: SCH 23390, a D1 receptor antagonist (10, 50 and 100 μM), PDTC (Pyrrolidinedithiocarbamate; 5 and 10 μM) and Sodium Salicilate (1 and 2 μM), inhibitors of NF-κB. SCH23390, PDTC and Sodium Salicilate were dissolved in PBS.

### Electrophoretic mobility shift assay (EMSA)

Nuclear extracts were prepared as previously described [30] with minor modifications. Briefly, cells were scraped in cold PBS (supplemented with 2 μg/mL leupeptin, 2 μg/mL antipain and 0.5 mM PMSF) and centrifuged at 4°C for 2 min at 13,000 g. Pellets were resuspended in lysis buffer (10 mM HEPES pH 7.9, 1.5 mM MgCl_2_, 10 mM KCl, 0.1 mM EDTA, 0.5 mM PMSF, 2 μg/mL leupeptin, 2 μg/mL antipain, 3 mM sodium ortovanadate, 30 mM sodium fluoride, 20 mM sodium pyrophosphate) and incubated on ice for 10 min. After the addition of NP-40 (to a final 0.5% concentration), samples were vigorously mixed and centrifuged for 30 s at 13,000 g. Supernatants were kept at -80°C for immunoblot analysis. Nuclei were resuspended in extraction buffer (20 mM HEPES, pH 7.9, 25% glycerol, 1.5 mM MgCl_2_, 300 mM NaCl, 0.25 mM EDTA, 0.5 mM PMSF, 2 μg/mL leupeptin, 2 μg/mL antipain), incubated for 20 min on ice and centrifuged for 20 min at 13,000 g at 4°C. The remaining supernatants containing nuclear proteins were stored at -80°C. Protein concentration was determined using the BioRad protein reagent. EMSA for NF-κB was performed using a gel shift assay kit from Promega. NF-κB double-stranded consensus oligonucleotide (5'-AGTTGAGGGGACTTTCCCAGGC-3') was end-labeled with γ-^32^P-ATP using T4 polynucleotide kinase. Unincorporated nucleotides were removed by passing the reaction mixture through a Sephadex G-25 spin column (Amersham-Pharmacia, Uppsala, Sweden). Purified ^32^P-labeled probe (30,000 cpm) was incubated in 20 μL with 5 μg nuclear extracts in a binding reaction mixture containing 50 mM NaCl, 0.2 mM EDTA, 0.5 mM DTT, 4% glycerol, 10 mM Tris-HCl (pH 7.5) and 0.05 μg poly (dI-dC) for 30 min at room temperature. DNA-protein complexes were separated by electrophoresis through a 6% non-denaturing acrylamide:bis-acrylamide (37.5:1) gel in 0.5× Tris-borate/EDTA (TBE) for 2 h at 150 V. Gels were vacuum-dried, and analyzed by autoradiography. For competition experiments, NF-κB and TFIID (5'-GCAGAGCATATAAGGTGAGGTAGGA-3') unlabeled double-stranded consensus oligonucleotide was included in 2-fold molar excess over the amount of ^32^P-NF-κB probe in order to detect specific and non-specific DNA-protein interactions, respectively. Unlabeled oligonucleotides were added to the reaction mixture 20 min before the radioactive probe. The composition of the complexes was determined by supershift assays; antibodies (1:10 dilution) against different NF-κB subunits (p50, p65, p52 and c-Rel) were added before the incubation of nuclear extracts with the labeled oligonucleotide. Autoradiographs were quantified by ChemImager detection system (Alpha-Innotech Corporation, USA).

### Immunoblot analyses

Electrophoresis was performed using a Bio-Rad mini-Protean II apparatus. In brief, the proteins present in the cytosolic (30 μg) fractions were size-separated in SDS-PAGE (10% or 15% acrylamide). The proteins were blotted onto a nitrocellulose membrane (Bio-Rad) and incubated with the specific antibodies (cytocrome-c sc-7159 (1:500) (Santa Cruz Biotechnology, CA, USA); α-spectrin MAB1522 (1:1000); IκB-α ab325 (1:250) Bax ab32503 (1:250), Bcl-2 ab7973 (1:250) (Abcam, Cambridge, MA, USA); or caspase-3 AB1899 (1:500) (Chemicon, Temecula, CA, USA). The Ponceau staining method was used to determine the loading amount (Salinovich and Montelaro, 1986). Immunoblots were quantified as described above. Proteins recognized by antibodies were revealed by ECL technique, following the instructions of the manufacturer (Amersham, Piscataway, NJ, USA). To standardize and quantify the immunoblots, a ChemImager detection system (Alpha-Innotech Corporation, USA) was used. β-actin antibody (sc-1616, Santa Cruz, CA, USA) was used as an internal control. Results were expressed in relation to the intensity of β-actin and as percentage of control value.

### Expression of Bax, Bcl-2 and BDNF

Total RNA was isolated using Trizol reagent and semi quantitative reverse transcription-PCR (RT-PCR) amplification was performed according to the instructions of the manufacturer (Invitrogen, Grand Island, NY, EUA). The primer sequences were: BDNF (304 bp), 5'-ATGCTCAGCAGTCAAGTGCC-3'(sense) and 5'-AGC CTTCCTTCGTGTAACCC-3'(antisense); Bax (271 bp), 5'-TGAACTGGACAACAACATGGAGC-3'(sense) and 5'-GGTCTTGGATCCAGACAAACAGC-3'(antisense); Bcl-2 (259 pb), 5'-GGAGGATTGTGGCCTTCTTTGAG-3' (sense) and 5'-TATGCACCCAGAGTGATGCAGGC-3' (antisense); GAPDH (258 pb), 5'-GCCAAGTATGATGACATCAAGAAG-3' (sense) and 5'-TCCAGGGGTTTCTTACTCCTTGGA-3' (antisense). The GAPDH was used for PCR control. The PCR conditions consisted of 45 s at 94°C, different number of cycles and temperature depending on the gene studied for 30 s (45 cycles, 55°C: BDNF; 28 cycles, 50°C: Bax; 40 cycles, 40°C: Bcl-2; 30 cycles, 50°C: GAPDH) and a final extension at 72°C for 10 min. Gel electrophoresis of the PCR product was performed using an ethidium bromide-containing agarose gel (1.5%), and resulting bands were visualized under UV light. The photographs were captured by VilberLourmat (Alpha-Innotech Corporation, EUA), and the optical density of the bands was determined using the ImageJ software. Results were expressed in relation to the intensity of GAPDH and as percentage of control value.

### Assessment of apoptosis by flow cytometry

DNA fragmentation was analyzed by flow cytometry after DNA staining with PI according to the method previously described [31]). After the incubation period, PC12 cells were removed from the dishes using trypsin/EDTA (2.5 g/L), centrifuged at 1000 × g for 15 min at 4°C, and the pellet was gently resuspended in 300 μL hypotonic solution containing 50 μg/mL PI, 0.1% sodium citrate, and 0.1% Triton X-100. The cells were then incubated for 2 h at 4°C. Fluorescence was measured and analyzed using a FACS Calibur flow cytometer (Becton Dickinson, San Juan, CA, USA. Fluorescence was measured using the FL2 channel (orange-red fluorescence = 585/42 nm). Ten thousand events were analyzed per experiment. Cells with PI fluorescence were then evaluated by using the Cell Quest software (Becton Dickinson), and results were expressed as a percentage of cells with DNA fragmentation in relation to untreated control cultures.

### MTT reduction and LDH release assay

Cell viability was estimated by the 3-[4,5-dimethylthiazol-2-yl]-2,5-diphenyl tetrazolium bromide (MTT) reduction assay [32]. After incubation of the cells with SCH 23390, PDTC, Sodium Salicilate and cocaine for 6 and 24 h, the culture medium was replaced by new one and MTT (final concentration 0.5 mg/mL) was added to the cells. The absorbance (570 nm) was measured after 30 min in a multiwell plate reader (Bio-tek instruments). To measure the release of lactate dehydrogenase (LDH) into the medium a colorimetric LDH-cytotoxicity assay kit was used (Roche Molecular BioChemicals, Indianapolis, IN, EUA). The results were expressed as percentage of control value at 490 nm determined after 15–20 min in a multiwell plate reader.

### Caspase 3 activity

To measure the effects of cocaine on caspase 3 activity colorimetric assay kit (Chemicon, Temecula, CA, USA) was used. After 6 hours of treatment with 1 mM cocaine cells were scraped in cold PBS, centrifuged at 1500 g for 10 minutes, resuspended in lyses buffer (50 mM Tris HCl, pH 7.4; 1 mM EDTA, 10 mM EGTA and 10 μM PMSF) and incubated for 10 minutes in ice. After centrifugation (10,000 g for 5 minutes) the supernatant was separated and incubated with 50 μL AC-DEVD-AMC (capase-3 substrate) at 37°C for 2 hours. 7-methylcoumarin (AMC), formed from the cleavage of the substrate by caspase 3, was spectrophotomteric assay at 460 nm. Increase in caspase activity was expressed as percentage of control values.

### Data analysis

Results were expressed as mean ± S.E.M. of the indicated number of experiments. Statistical comparisons were performed by non-paired Student's t test and one-way ANOVA followed by the Newman-Keuls test P < 0.05 was considered as statistically significant. All statistics analyses were performed using a Prism4 software package (Graphpad Software, San Diego, CA, USA).

## Results

### Cocaine caused a time and concentration dependent activation of NF-κB in PC12 cells

**Figure 1:**
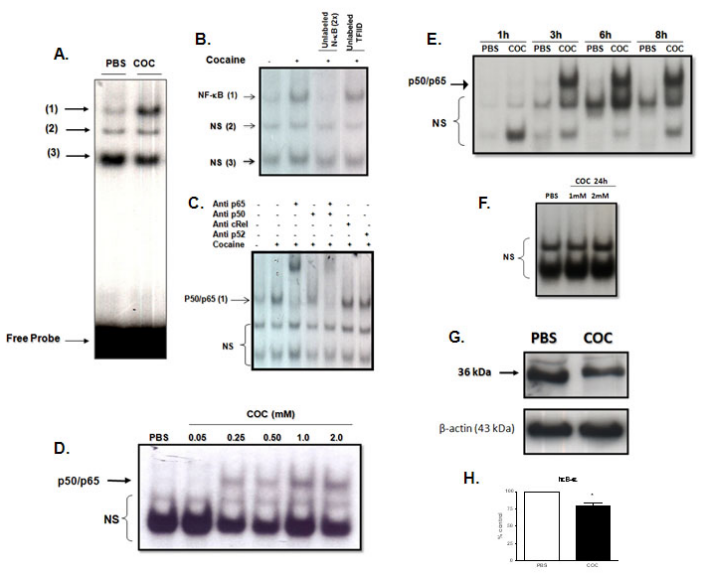
**PC12 cells were treated with 1 mM cocaine (COC) or PBS for 6 hours**. Nuclear extract (5 μg) was submitted to EMSA assay. (A) Positions of protein/DNA complexes observed are indicated by numbers. Localization of free probe is also indicated. (B) Competition studies in the absence or presence of unlabeled specific (NF-κB consensus sequence 2-fold molar in excess) or non-specific oligonucleotide (TFIID consensus sequence, 2-fold molar in excess), as indicated. (C) Supershift assays incubated in the absence or presence of 2 μL of antibodies (1:10 dilution) against p50, p65, c-Rel and p52 subunits, as indicated. Antibodies were added 20 min prior to addition of the radiolabeled NF-κB consensus oligonucleotide. The position of specific NF-κB/DNA binding complex p50/p65 (band 1) is indicated. NS represents no specific binding (bands 2 and 3). (D) Concentration response curve and time-course of NF-κB activation induced by COC in PC12 cells. Nuclear proteins (5 μg) were extracted from cells treated with different concentrations of cocaine: 0.5, 0.25,0.5, 0.75, 1, 2 mM or PBS for 6 hours (E) Different time points of NF-κB activation: 1, 3, 6 and 8 hours with 1 mM of COC (panel B) or (F) 24 hours with 1 and 2 mM COC or PBS. The position of specific NF-κB/DNA binding complex (p50/p65 complex) is indicated. (G) Representative immunoblot expression of IκB-α in PC12 cells after incubation with COC (1 mM) or PBS for 6 hours. The densitometric analysis (% of control) of the IκB-α band complex represented in (G) is shown (H). β-Actin was used as internal control. Results are expressed as mean + S.E.M. from three individual experiments. * p < 0.05 vs PBS (Student's *t*-test).

Nuclear extracts from cells treated with 1 mM cocaine for 6 hours presented three DNA/protein complexes indicated by EMSA assay (Figure 1A). The complex 1 was displaced by an excess of unlabeled NF-κB, but not by TFIID double stranded oligonucleotide consensus sequence, demonstrating the specificity of NF-κB/DNA binding interaction of this complex (Figure 1B). Complexes 2 and 3 were not displaced by the unlabeled NF-κB and are not considered to be associated to NF-κB family.

Supershift analysis indicated that the p65 subunit antibody shifted DNA/protein interactions found in complex 1. The p50 subunit antibody induced a partial decrease in complex 1. In contrast, the antibodies against the p52 and c-Rel subunits did not affect DNA-protein complex (Figure 1C). This suggests that p50/p65 heterodimers were included in ^32^P-NF-κB/protein complex 1. Complexes 2 and 3 were not displaced by the antibodies confirming that they are not related to NF-κB family. Because complex 1, mainly composed by p50/p65 heterodimers, was the major DNA/protein complex altered by the treatments, the term NF-κB was used to identify this complex.

PC12 cells were incubated for 6 hours with different concentrations of cocaine (0.05 – 2.0 mM) and nuclear proteins were submitted to EMSA assay. Activation of NF-κB by cocaine was concentration-dependent; starting at 0.25 mM and with maximum response at 1 mM (Figure 1D). We also observed that the activation of NF-κB started after 3 hours of treatment and persisted until to 8 hours (Figure 1E), however, 24 hours latter (Figure 1F) there was no activation of NF-κB. The concentration of 1 mM cocaine and 6 hours of incubation were then used to evaluate changes induced by cocaine on NF-kB pathway in PC12 cells.

### Increase of IκB-α degradation caused after cocaine exposure

To confirm the activation of NF-κB by cocaine in PC12 cells the content of IκB-α in the cytoplasm of cells treated with 1 mM cocaine for 6 hours was measured. A reduction in IκB-α expression (Figures 1G and 1H) was observed, confirming our previous data.

### Involvement of the D1 receptor in the effects of cocaine-induced NF-κB binding activity

**Figure 2:**
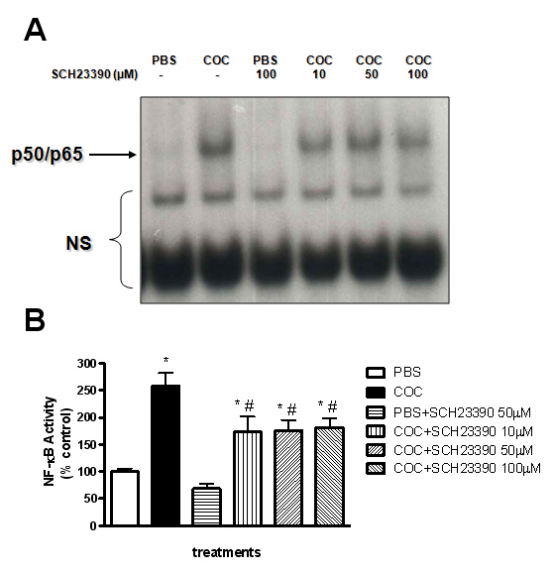
**Influence of SCH23390 on NF-κB activation induced by cocaine in PC12 cells**. Cells treated with SCH23390 (10, 50, 100 μM) 20 min prior to cocaine (COC) (1 mM) or PBS treatments were incubated for 6 hours and EMSA assay was performed with the nuclear extract. Panel A represents autoradiography of the EMSA assay. The position of specific NF-κB/DNA binding complex p50/p65 is indicated. Bottom graphic (panel B) represents densitometric analysis of the p50/p65 bands presented in the top panel. Results are expressed as mean + S.E.M. from three individual experiments. * p < 0.05 vs PBS and # p < 0.05 vs COC (one-way ANOVA followed by Newman-Keuls test).

As cocaine indirectly induces activation of D1 receptors, the cells were pre-treated with a D1 antagonist, SCH 23390, twenty minutes before the exposure to cocaine (1 mM). SCH 23390, at all concentrations tested, partially inhibited the activation of NF-κB by cocaine (Figure 2), as concluded by a partial reduction in the quantification of NF-κB band compared to cocaine treated cells alone.

### Cocaine exposure induced PC12 cell death

Three methods (FACS, MTT and LDH activity) were used to evaluate PC12 cell death. All assays were performed after 6 and 24 hours of incubation with 1 mM cocaine.

**Figure 3:**
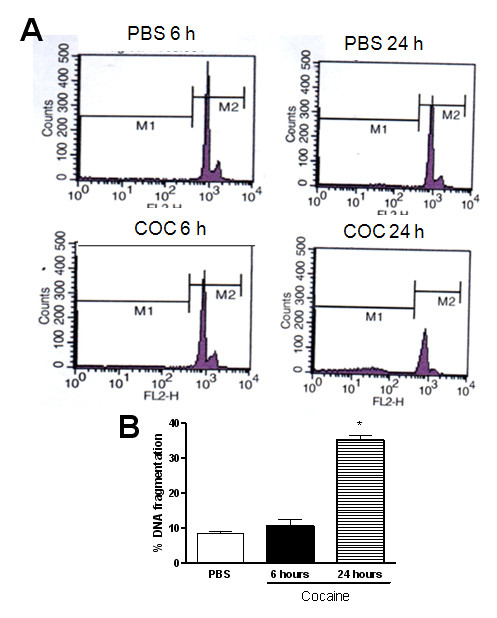
**Effect of cocaine (COC) (1 mM, 6 hours) on DNA fragmentation in PC12 Cells**. A flow cytometric analysis was conducted after the cells had been incubated for 6 and 24 hours with cocaine. Results are expressed as mean + S.E.M. from three individual experiments. * p < 0.05 vs PBS (one-way ANOVA followed by Newman-Keuls test).

Flow cytometric analysis was used to evaluate the effects of cocaine on DNA fragmentation in PC12 cells. The proportion of apoptotic cells in cocaine-treated PC12 cells (1 mM, 24 hours) was approximately 25-fold greater than in control cells (PBS-treated) (Figure 3). No change was observed after 6 hours.

**Figure 4:**
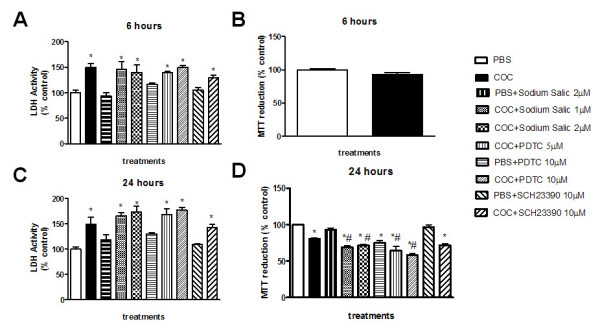
**Effect of PDTC, Sodium Salicilate and SCH23390 on cocaine-Induced toxicity in PC12 Cells (A and C: LDH release assay; B and D: MTT reduction assay)**. PC12 cells were pre-treated for 20 min with various concentrations of PDTC, Sodium Salicilate or SCH23390 prior to cocaine (1 mM, 6 hours) treatment. Cell viability was estimated by MTT reduction (570 nm) and LDH release (490 nm) assays. Results are expressed as mean + S.E.M. from three individual experiments. * p < 0.05 vs PBS and # p < 0.05 vs COC (cocaine) (one-way ANOVA followed by Newman-Keuls test)

The MTT assay indicates changes in metabolic activity associated to cell survival (cell viability). A decrease in metabolic activity of PC12 cells treated with cocaine for 24 hours (Figure 4D) was observed as compared with controls, indicative of overall decreased cell viability. No change was observed after 6 hour treatment (Figure 4B).

LDH is released from cells as a result of loss of plasma membrane integrity. Incubation with cocaine raised LDH activity in the medium after 6 (Figure 4A) and 24 hours (Figure 4C).

### Antagonism of the D1 receptor did not change cell viability

The effects of SCH 23390 on cocaine-induced PC12 cell death were evaluated by measuring MTT reduction and LDH release to the incubation medium. The pre-treatment of the cells with 10 μM SCH 23390 did not change cell viability (Figure 4).

### Inhibitors of NF-κB increased PC12 cell death caused by cocaine

We next determined whether PDTC or Sodium Salicilate modulates cocaine-induced cell toxicity as assessed by LDH and MTT assays. Sodium salicilate inhibits the IκB-α degradation, preventing the translocation of NF-κB to the nucleus (Koop and Ghosh, 1994), whereas PDTC directly inhibits NF-κB. Both drugs partially decreased NF-κB activation by cocaine (data not shown). The decrease in PC12 cell viability induced by cocaine was more pronounced in the presence of the NF-κB inhibitors (Figure 4D). No difference was seen in the LDH assay (Figures 4A and 4C).

### Cocaine exposure increased the α-spectrin cleavage

**Figure 5:**
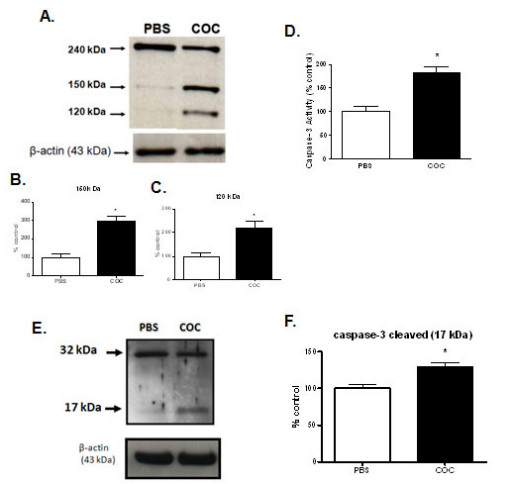
**(A) Cocaine (COC) exposure increased α-spectrin (240 kDa) cleavage and (E) increased cleavage of caspase 3 in PC12 cells**. Cell lysates were extracted from PBS and cocaine (1 mM, 6 hours) treated PC12 cells. Cell lysate protein content (30 μg) was loaded and run on a SDS-polyacrylamide gel and submitted to Immunoblot assay. The densitometric analysis (% of control) of the 150,120 kDa (cleaved product of spectrin) and caspase-3 cleaved product (17 kDa) band complexes represented in A and D are shown on B, C and F respectably. β-Actin was used as internal control. (D) COC exposure raised caspase-3 activity and expression in PC12 cells. Cells were treated with 1 mM of COC for 6 hours. Cell lysates were analyzed for caspase-3 activity, using the caspase-3 substrate (Ac-DEVD-AMC). Results are expressed as mean + S.E.M. from three individual experiments; * p < 0.05 vs PBS (Student's *t*-test).

Spectrin plays a key role to maintain cellular membrane proprieties. Cocaine (1 mM) augmented α-spectrin fragmentation (150 e 120 kDa) after 6 hours incubation (Figures 5A, B and 5C). This fragmentation was not observed in PBS-treated cells. Activation of calpain and/or caspase-3 resulted in α-spectrin cleavage to 150 and 120 kDa fragments by caspase and 150 and 145 kDa by calpain. An increase in the 150 and 120 kDa fragments indicating that caspase-3 plays an important role in cell death induced by cocaine.

### Cocaine activated caspase-3 in PC12 cells

Cocaine-induced α-spectrin fragmentation and other reports suggesting that caspase-3 is the major protease in apoptosis led us to verify whether caspase-3 activation was involved in the induction of PC12 cells apoptosis by cocaine. Caspase-3 activity was measured at 6 h, a critical time point at which cocaine induced the highest NF-κB activation. Cocaine increased caspase-3 activity as well as protein cleavage (another index of caspase-3 activation) in PC12 cells when compared to control (PBS-treated) cells (Figures 5D, E and 5F).

### Cocaine reduced Bcl-2 in PC12 cells

**Figure 6:**
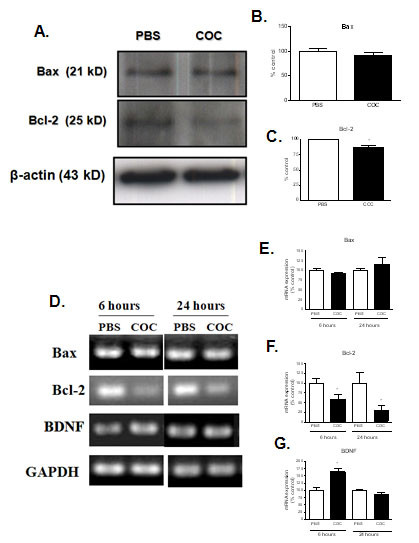
**(A) Cocaine (COC) exposure decreased Bcl-2 protein expression in PC12 cells**. Cell lysates were extracted from PBS and COC (1 mM, 6 hours) treated PC12 cells. Cell lysate protein content (30 μg) was loaded and run on a SDS-polyacrylamide gel and submitted to Immunoblot assay. The densitometric analysis (% of control) of the Bax and Bcl-2 band complexes represented in (A) are shown (B and C). β-Actin was used as internal control. (D) Effect of incubation with COC (1 mM) for 6 or 24 hours on levels of mRNA for Bax, Bcl-2 and BDNF in PC12 cells. Cocaine increased BDNF mRNA levels after 6 hours (G) and decreased Bcl-2 mRNA levels after 6 and 24 hours of cocaine treatment (F) as indicated by RT-PCR assays. No changes were observed for Bax mRNA levels after both 6 and 24 hours of cocaine treatment (E). Results are expressed as mean + S.E.M. from three individual experiments. * p < 0.05 vs PBS (one-way ANOVA followed by Newman-Keuls test).

Cocaine exposure significantly reduced Bcl-2 mRNA and protein levels after 6 hour exposure and the mRNA levels were still lower after 24 hours. No changes in Bax mRNA and protein levels were observed (Figure 6).

### Cocaine transiently increased BDNF mRNA levels

Since BDNF (Brain Derived Neurotrophic Factor) is a neurotrofin that participates in the neuronal protection and its expression can be regulated by transcription factors (such as NF-κB) the expression of this protein was investigated in PC12 cells after incubation with cocaine. We observed an increase in BDNF mRNA levels after 6 hours of treatment, which was not seen after a longer period (24 hours) of incubation (Figure 6F).

## Discussion

Cocaine treatment induced PC 12 cell death by apoptosis and necrosis. These effects were verified by mitochondrial dysfunction, increase in LDH release, activation of caspase 3, decrease in Bcl-2 expression and increase in α-spectrin cleavage. Cocaine treatment also activated the p50/p65 subunit of NF-κB in PC 12 cells after 6 hour partially due to the activation of D1 dopamine receptor.

Cocaine concentrations used in this study were similar to those previously employed by others in different cell types [18,33-36]. A wide range of cocaine plasma levels (0.3 μM to 1 mM) has been reported in subjects who use this drug [34,37] being compatible with the concentration used in this study.

Cocaine treatment activated NF-κB in PC12 cells in the interval of 3–8 hours while cell death was more pronounced after 24 hours only. Supershift assay analysis indicated that cocaine increased the p50/p65 NF-κB complex content. In agreement with our results, others showed activation of NF-κB by cocaine in different models. Chronic administration of cocaine induced NF-κB activation in nucleus accumbens of mice [38]. Cocaine-induced NF-κB activation was also observed in macrophages [39], human brain endothelial cells [40], and in PC12 cells [17]. Imam et al. [17] showed that 24 hours of exposure to low concentrations of cocaine (5–500 μM) caused an increase of NF-κB activity in PC12 cells and, in agreement with our results, higher concentrations of this drug did not show significant alteration of this transcription factor after 24 hour treatment.

So, none of these studies investigated the mechanism of cocaine-induced NF-κB activation. This is the first report that investigates the mechanism linking activation of NF-κB and cell death induced by cocaine.

The involvement of dopaminergic receptors in the activation of this transcription factor by cocaine was also investigated in PC 12 cells. Pre-incubation with a D1 antagonist, SCH 23390, caused a partial reduction in cocaine-induced NF-κB activation, suggesting the participation of these receptors in this process. Others have also investigated the participation of dopamine receptors in the activation of NF-κB. Dopamine D1 receptor raised the expression of immediate early genes (such as, c-fos, c-jun, junB and zif-268 [41]) that act as positive modulators of transcription factors, including NF-κB [42-44].

Han and col. (2007) suggested that the increase in NF-κB activity caused by dopamine occurs by its interaction with D1 receptors leading to the activation of G protein, increased levels of intracellular cyclic AMP and stimulation of the phospholypase C/PKC pathway [45]. These events would lead to the activation of MAP kinases, which in turn could activate NF-κB. Yang et al. (2003) reported that in human cervical carcinoma (HeLa) cells transiently expressing human D2 receptors, dopamine-induced activation of NF-κB is mainly dependent upon c-Src activation [46]. These investigators also presented evidence that the PI3K and the MEK-ERK pathways are involved in dopamine-induced activation of NF-κB.

Dopamine readily oxidizes to form reactive oxygen species (ROS), free radicals, and quinones, a process that can occur either spontaneously in the presence of transition metal ions [47] or via an enzyme catalyzed reaction [48]. Activation of dopamine receptor can also increase peroxide hydrogen (H_2_O_2_) formation by protein kinase C (PKC), subsequently leading to activation of p38 MAPK and JNK, that stimulate NF-κB activation as showed by Lee et al. (2006) in mouse embryonic cells [49]. In the neuronal cell line, NG108-15, the D2 receptor-induced NF-κB activation depends upon the MEK-ERK pathway, rather than p38 MAPK [50]. Therefore, multiple mechanisms have been reported to be responsible for mediating the effects of dopamine on NF-κB activation in different cell types.

NF-kB is a transcription factor activated in response to cellular stress that is involved in the regulation of apoptosis. Depending on the cell type and the apoptotic agent, NF-κB has been reported to mediate or prevent apoptosis [51]. The cell death process induced by cocaine was initiated at 6 hours being more pronounced after 24 hours of exposure. We also observed that both necrosis and apoptosis are involved in the induction of cell death by cocaine as observed by rupture of the cell membrane and DNA fragmentation in agreement with finding by others [17,19,52].

To further investigate the role of NF-κB in cocaine induced-cytotoxicity NF-κB cell permeable inhibitors were used. The inhibition of NF-κB significantly increased the cell death promoted by cocaine treatment, suggesting that this transcription factor plays a protective role in cocaine treated PC12 cells. Lee and colleagues showed an anti-apoptotic effect of NF-κB in PC12 cells death induced by auto-oxidized dopamine [40]. High constitutive NF-κB activity mediates resistance to oxidative stress in neuronal cells [53] and, agents that inhibit NF-κB activation induced apoptosis in response to several neurotoxins [54-58].

Cocaine caused a reduction in anti-apoptotic Bcl-2 protein level but no change in pro-apoptotic Bax altering, therefore, the Bax/Bcl-2 ratio, which triggers cellular apoptosis. The reduction of Bcl-2 levels after exposure to cocaine has also been reported in fetal rat myocardial cells [59]. However an increase in Bax levels was observed after 30 minutes and 1 hour of exposure to cocaine (1.5 μM) in locus coerulus cells [36], whereas no change was found herein. These results demonstrate that different concentrations of cocaine and time of exposure can differently regulate the Bax/Bcl-2 ratio.

In addition, study of caspases, which are also important regulators of apoptosis, revealed that exposure of PC12 cells to cocaine increased caspase-3 activity of and the cleavage of caspase-3 precursors. Several studies have reported the induction of caspase-3 by cocaine both in *vitro *[16,18,36,60] and *in vivo *[15,61]. Cocaine also caused α-spectrin cleavage in fragments that correspond to the degradation mediated by caspase-3, suggesting that calpain is not involved in the α-spectrin fragmentation process induced by this drug [62]. This data, together with the increase observed in the caspase-3 activity, suggest that cocaine induced apoptosis in PC12 cell by caspase-3 activation.

An increase of BDNF mRNA levels was found after 6 hours of treatment with cocaine only, indicating a transitory augment of this neurotrophin. Le Foll and colleagues also observed a transitory increase of BDNF mRNA levels in frontal cortex of rats after a single injection of cocaine [63]. BDNF regulates the differentiation and apoptosis of neurons and glial cells [64], and the increase in BDNF may be considered as a line of defense against the apoptosis process caused by cocaine in our model. In fact, the increase in BDNF mRNA levels could be linked to the activation of NF-κB [65]. The protective role of NF-κB in cocaine treatment of PC12 cells observed herein may be associated to expression of anti-apoptotic genes, such as BDNF. However the compensatory mechanisms to cell death induced by cocaine were ineffective to abolish the apoptosis process.

## Conclusion

**Figure 7:**
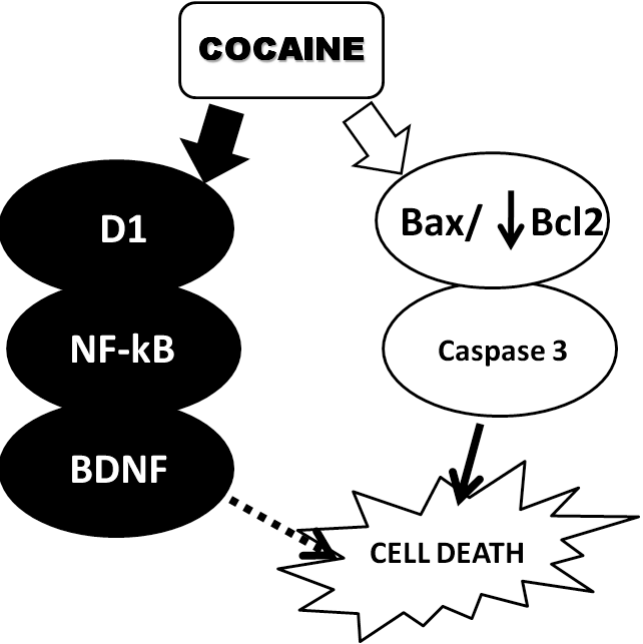
**Schematic representation of neurotoxic action of cocaine in PC12 cells**. The treatment of PC 12 cells with cocaine can alter the Bax/Bcl-2 ratio, reducing the Bcl2 levels, which could lead to activation of caspase 3 and the trigger of cell death process, seen after 24 hours of treatment. On the other hand, to protect the cell from cocaine-toxic effects activation of NF-κB occurs through D1 receptors. The activation of this transcription factor could lead to transcription of anti-apoptotic genes, such as BDNF that tries to mimitaze the process of cell death.

The exposure of PC12 cells to cocaine decreased the ratio Bax/Bcl-2, reducing the levels of Bcl-2, and leading to activation of caspase-3 that can act as a modulator of the apoptosis process observed after 24 hours of exposure to the drug (Figure 7). Moreover, cocaine activated NF-κB (p50/p65) in a concentration and time dependent manner and this effect was partially mediated by D1 receptors. The activation of this transcription factor may represent a compensatory mechanism to limit cell death.

## Competing interests

The author(s) declare that, except for income received from my primary employer, no financial support or compensation has been received from any individual or corporate entity over the past three years for research or professional service and there are no personal financial holdings that could be perceived as constituting a potential conflict of interest.

## Authors' contributions

LBL: participated in all of the experiments, have made substantial contributions to conception and design, acquisition of data, analysis and interpretation of data; have been involved in drafting the manuscript or revising it critically for important intellectual content; and have written the manuscrip to be published.

CDM: have made substantial contributions to conception and design, acquisition of data, analysis and interpretation of data; have been involved in drafting the manuscript or revising it critically for important intellectual content, have given final approval of the version to be published.

EMK: have made substantial contributions to conception and design, acquisition of data, analysis and interpretation of data; have given final approval of the version to be published.

LMY: participated in the design of the study and performed the statistical analysis; have given final approval of the version to be published.

LSL: particpated in the performance of the experimental (cell culture, Gel Shifts assay)

MFB: carried out the FACS assay and have given final approval of the version to be published.

TML: carried out the FACS assay and have given final approval of the version to be published.

RC: participated in the design of the study and performed the statistical analysis; have given final approval of the version to be published.

CSP: have made substantial contributions to conception and design, acquisition of data, analysis and interpretation of data; have been involved in drafting the manuscript or revising it critically for important intellectual content, have given final approval of the version to be published.

CS: have made substantial contributions to conception and design, acquisition of data, analysis and interpretation of data; have been involved in drafting the manuscript or revising it critically for important intellectual content, conceived of the study, and participated in its design and coordination.

## Authors' information

This work is part of studies regarding the PhD thesis of LBL.
